# The Moderating Effect of Employee Political Skill on the Link between Perceptions of a Victimizing Work Environment and Job Performance

**DOI:** 10.3389/fpsyg.2017.00850

**Published:** 2017-05-30

**Authors:** Jeffrey R. Bentley, Darren C. Treadway, Lisa V. Williams, Brooke Ann Gazdag, Jun Yang

**Affiliations:** ^1^Department of Management and Human Resource Management, California State University, Long Beach, Long BeachCA, United States; ^2^Organization and Human Resource Department, University at Buffalo, State University of New York, BuffaloNY, United States; ^3^Department of Management, Niagara University, LewistonNY, United States; ^4^Institute for Leadership and Organization, Ludwig-Maximilians-Universität MünchenMunich, Germany; ^5^Department of Management, East Carolina University, GreenvilleNC, United States

**Keywords:** victimization, political skill, job performance, conservation of resources, stress, tension

## Abstract

Research has generally revealed only a weak link, if any at all, between victimization-related experiences and job performance. Drawing on the commonly used conservation of resources perspective, we argue that such inconsistent evidence in the organizational literature stems from an over-focus on personal resources at the expense of considering the role of social resources. Victimization is an interpersonal phenomenon with social ramifications. Its effects may be better captured when measured from the standpoint of the social environment, and analyzed relative to an employee’s capacity to effectively regulate those social resources. With the latter capacity being encapsulated by the construct of political skill, we conducted two studies to explore the moderating influence of employee political skill on the relationship between employee perceptions of a victimizing work environment and employee task performance. In Study 1, employees with low political skill exhibited reduced task performance when perceiving a victimizing environment, and this link was found to be mediated by tension in Study 2. Those with high political skill exhibit no change in performance across victimization perceptions in Study 2, yet an increase in performance in Study 1. We discuss our findings relative to the victimization and political skill literatures.

## Introduction

Professionals in the workplace have recently grown interested in reducing the harmful effects of workplace victimization on employee performance (e.g., Stevens, 2002; Know Bull!, 2010; Workplace Bullying Institute, 2014). Organizational researchers, on the other hand, are only beginning to explore this problem. Workplace victimization and similar harassment-related experiences, such as bullying or abuse, have been strongly linked to distress and strain (Aquino and Thau, 2009), as well as retaliation against aggressors (Andersson and Pearson, 1999; Lian et al., 2014). Empirical work, however, has only found an inconsistent link between victimization and performance (Bowling and Beehr, 2006). Moreover, our understanding of the types of employees who thrive despite the presence of victimization has yet to be refined. In working to resolve this gap, we consider a critical aspect of the victimization process that has not yet been extended to our understanding of its consequences: social resource regulation.

Workplace victimization is as much a social phenomenon as it is a personal one. Employees’ social resources, such as status (Aquino et al., 1999) or power (Aquino and Bradfield, 2000), for instance, have been linked to their likelihood of perceiving victimization, while social perceptions (e.g., victim vs. victimizer role prototypes) are believed to be a critical mechanism in sustaining the victimization process once initiated (Aquino and Lamertz, 2004). Because performance behavior is also highly tied to social interactions in the broader work environment (Gerstner and Day, 1997; Sparrowe et al., 2001; Thompson, 2005), it stands to reason that employees’ perceptions of victimization toward themselves *as well as toward their peers* in their socio-relational work environment will affect their performance. The extent of this effect, however, would necessarily rely on whether or not employees are able to effectively manage their social resources to avoid the stress and strain that comes from resource loss.

**FIGURE 1:**
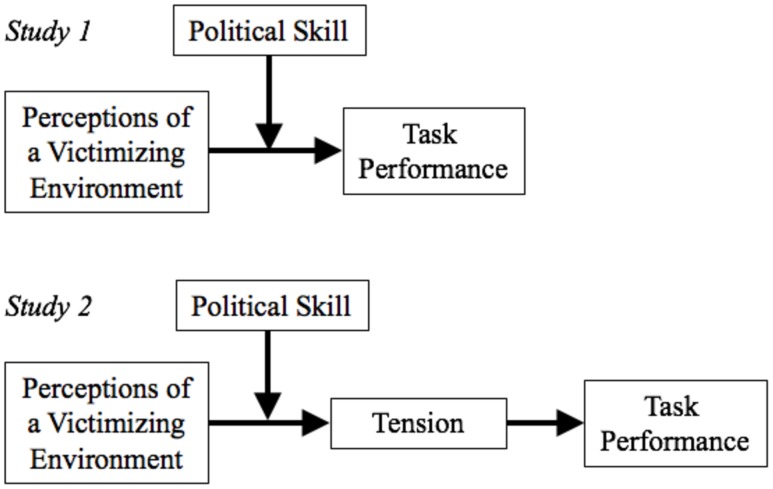
**Conceptual models for Studies 1 and 2**.

Leveraging the Conservation of resources (COR: Hobfoll, 1989, 2002) perspective on stress, we suggest that perceiving victimization in one’s socio-relational work environment triggers systemic changes in how employees regulate their social resources, toward the goal of preserving as many as possible in the face of depletion. The effectiveness of these changes depends on the efficiency with which employees are able to both recognize the state of present resources and potential for future social resources, as well as to influence others in ways that protect those resources and the likelihood of future accumulation. In organizational research, political skill (Ferris et al., 2005) is a primary exemplar of this capacity, as such we expect that those with high political skill will be effective in managing social resources to benefit their job performance. Through a two-study replication, we first test the interactive effects of perceiving a victimizing environment and political skill on task performance. We then further test the underlying resource depletion model in a second study by determining the extent to which political skill results in reduced tension (which signifies reduced resource depletion) in response to perceiving a victimizing environment, and how that affects job performance (**Figure 1**).

The present study contributes toward efforts to both understand and manage the rise of workplace victimization. In regards to theory, we expand upon existing COR-based models of victimization by illuminating the role of social resource management on resource depletion and performance, rather than just social resources in isolation (e.g., status: Aquino et al., 1999). Next, this study offers evidence that social resource regulation may be another critical pathway through which strain and resource depletion affects employee behavior, in complement to extant models surrounding self-regulatory failure (Thau and Mitchell, 2010; Lian et al., 2014) and social exchange violation (Cropanzano et al., 2003). Lastly, we extend the domain of workplace stressors that employee political skill has been found to mollify to include victimization. In terms of practical significance, by identifying political skill as a personal characteristic that neutralizes the deleterious effects of victimizing environments on performance, we highlight a trainable means (Ferris et al., 2007) through which organizations can prevent the negative performance effects of the downward spiral (e.g., Andersson and Pearson, 1999) of victimization.

## Workplace Victimization and Job Performance

Workplace victimization affects up to 48% of employees yearly, with 27% being bullied, 21% witnessing bullying (Zapf et al., 2003; Workplace Bullying Institute, 2014), and with 45% in some samples reporting at least one victimizing act a week (Lutgen-Sandvik et al., 2007). Defined as “an individual’s self-perception of having been exposed, either momentarily or repeatedly, to aggressive actions emanating from one or more other persons” (Aquino, 2000, p. 172), workplace victimization includes other forms of intentionally harmful behavior, such as bullying, harassment, and abusive supervision (Bowling and Beehr, 2006; Tepper and Henle, 2010). Research on victimization-related experiences in the workplace have found weak to moderate, yet statistically significant, links between such experiences and behavior like job performance or deviance (Aquino and Thau, 2009; Hershcovis and Barling, 2010). Most arguments for the effects of victimization-related experiences on work behavior in general are based on Hobfoll’s (1988, 1989, 2002) COR perspective common in the stress literature.

Conservation of resources proposes that people associate various resources with themselves, including physical objects, conditions (e.g., tenure, job advancement), feelings, personal characteristics, and energies (e.g., knowledge, money). Hobfoll’s (1988) fundamental premise is that people experience stress when their resources are threatened with loss, actually lost, or not regained after expenditure, and that people are instinctively driven to minimize resource loss by using as few resources as possible. If this stress goes on for too long it can turn into strain and impair psychological health. Many studies use the resource depletion argument and find evidence that victimization-related behavior enhances strain or emotional exhaustion (Winstanley and Whittington, 2002; Bowling and Beehr, 2006; Grandey et al., 2007), which then affects behavior by redirecting attention toward the offending stimuli, impairing self-control (Thau and Mitchell, 2010; Lian et al., 2014), or by creating a sense of vulnerability that reduces employees’ motivation to reciprocate to the organization (Cropanzano et al., 2003; Chang et al., 2007).

Resource depletion studies generally explore the effects of discrete resources on the arising of victimization-related experiences, or characteristics affecting employee reactions to personal resources. Aryee et al. (2010), for instance, conceptualize work structures (organic vs. mechanistic) as a resource and find that organic work structures neutralize the negative effect of abusive supervision on contextual performance. In examining reactions to depletion, Wheeler et al. (2013) find that employees with higher psychological entitlement often become emotionally exhausted in response to abusive supervision and in-turn abuse their co-workers, in theory because of their poor capacity to regulate their emotional resources. Among these studies, those that link victimization-related behavior to job performance often have the weakest effect sizes and are inconsistent in finding support (Bowling and Beehr, 2006). While these studies have dealt with the ramifications of structural and personal resources, their findings may be inconsistent because they neglect a fundamental aspect of victimization, in terms of both conceiving its effects as well as the characteristics that alter depletion, the social ramifications of being victimized.

### Social Resources and Social Perception in Workplace Victimization

What is often over-looked in the COR model is Hobfoll’s (1988) detailed assertion that through physiological, cognitive, and non-conscious means, resources can be evaluated and transformed into new resources that take on greater meaning. Status, for instance, which is a social resource, can be earned by developing one’s personal characteristics, whereas owed-reciprocity (an aspect of social capital) can come from optimistically (i.e., a feeling) investing time and knowledge (i.e., energies) to benefit others. Over the past decade, research on victimization by Aquino and colleagues has progressively uncovered the effects of social perceptions and social resources on victimization, especially relative to both personal resources and interactions with others. Aquino et al. (1999), for instance, found that employee negative affectivity was less likely to enhance victimization perceptions if they held high status, while Aquino et al. (2001) found those with higher status less likely to engage in retaliation when they blamed others for personal offenses; the researchers suggested these arise from greater perceived power to control processes, rewards, and retaliation. Similarly, Aquino and Bommer (2003) found that performing helping behavior also reduced perceived victimization. In this case, the researchers suggested that helping heightens the performer’s social value by enhancing the perception that one is obligated to reciprocate and protect the performer.

In complement to social resources, Aquino and Lamertz (2004) explored the importance of social perceptions in the victimization process. They reviewed evidence and proposed that employees engage in behavioral patterns that are characteristic of either a victim or victimizer role archetype, which then encourages others in the environment to respond accordingly (e.g., enacting the Provocative Victim role will elicit aggressive force by those who recognize and engages in the relational role-set). Once engaged, employees are constrained to act increasingly in line with the social expectations of their role as a victim or victimizer. Thus, where social resources affect the rise of victimization, the maintenance of victimization may depend on a larger range of social perceptions (e.g., norms, expectations, attributions) reinforced in the relational environment. Past research, then, may have failed to capture the effects of victimization on performance because it was not capturing the threat of victimization from a social standpoint rather than a personal one. A more inclusive measure of victimization may need to consider employee perceptions of victimization in the work environment as a whole; we advance such a position below and describe its impact on job performance.

## Perceptions of a Victimizing Environment, Political Skill, and Job Performance

Evidence from the practical world of work links perceptions of a victimizing environment to social repercussions and productivity issues. At the Tribune Company, for instance, ongoing use of “sexual innuendo, poisonous workplace banter and profane invective” (Carr, 2010: p. 1) in the culture “offended many employees” (Arango and Carr, 2010, p. 1) and coincided with declining employee performance and pending bankruptcy. Whereas in healthcare, medical students (Sheehan et al., 1990; Fried et al., 2012) and nurses (Lewis, 2006; Roberts et al., 2009) who normatively experienced verbal abuse and humiliation, often in the presence of peers, reported lowered confidence and higher feelings of social alienation that affected them negatively throughout their time in the organization.

Victimization, in its very nature, threatens resources employees associate with themselves, such as emotional positivity or health, and has indeed been linked with stress (e.g., Buss, 1961; Lewis and Orford, 2005; Bowling and Beehr, 2006; Aquino and Thau, 2009). When considering only oneself, victimization threatens personal resources, such as feelings or self-esteem. In such cases, avoidance or retaliation can stem resource loss (for review see Aquino and Thau, 2009). Yet when *perceived diffusely in one’s social environment*, the potency of various relationally based resources (e.g., reputation, relationships) falls under threat as well. Indeed, research suggests that victims feel vulnerable to attack from others if victims believe they are losing status (Perloff, 1983; Aquino, 2000). In their study of social undermining at work, Duffy et al. (2002) posited that intentional harm received from others deteriorates one’s effectiveness in managing social relations, and found evidence that experiencing undermining behavior reduced self-efficacy and physical health, and heightened counterproductive behaviors.

Victimization, then, may most strongly affect behavior when perceived broadly in the environment, rather than when perceived in unique dyadic interactions. Perceiving a victimizing work environment can specifically impair performance because the latter is also embedded within the social perceptions and resources in the relational environment at work. Job performance often relies on accumulating and leveraging one’s personal resources (e.g., self-esteem, self-efficacy; Tharenou and Harker, 1982; Leary et al., 1995; Stajkovic and Luthans, 1998) and social resources (e.g., social capital; Sparrowe et al., 2001; Thompson, 2005) in the broader social-relational environment via social connections and social resource exchange (e.g., Wood and Bandura, 1989; Gerstner and Day, 1997). In light of this, employees’ capacity to leverage and regulate their social resources is critical in determining their performance when they perceive a victimizing environment; that capacity in the workplace is known as political skill.

### Political Skill as an Antidote to Perceptions of a Victimizing Environment

As “the ability to effectively understand others at work and to use such knowledge to influence others to act in ways that enhance one’s personal and/or organizational objectives” (Ferris et al., 2005, p.127), political skill plays a large role in determining an employee’s success in enacting social influence behavior in the workplace (Harris et al., 2007; Kolodinsky et al., 2007; Treadway et al., 2007). Rooted in deep-level psychological traits (e.g., self-monitoring, positive affectivity, conscientiousness, proactivity, etc.; Ferris et al., 2007, 2008), Ferris et al. (2005) find that political skill consists of four interrelated dimensions: social astuteness, interpersonal influence, networking, and apparent sincerity. Combined, these capacities enhance politically skilled employees’ sensitivity to social cues (social astuteness) and allows employees to adapt their behavior accordingly (interpersonal influence), appearing self-confident, and genuine (apparent sincerity), and developing resource-rich social networks (networking ability).

From the COR perspective, political skill can be conceived as a personal resource (Hochwarter et al., 2006) letting employees achieve goals by effectively leveraging personal and social resources; it is a personal resource that facilitates protection of social resources. When faced with stressors, politically skilled employees do not experience strain reactions as strongly as less the politically skilled workers (Perrewé et al., 2004), in theory, due to the formers’ enhanced sense of security and control over their social environment (Perrewé et al., 2000; Ferris et al., 2007). Being socially astute (Ferris et al., 2005, 2007), the politically skilled are more likely than less skilled employees to understand the social implications of resource expenditures, discern the present and potential value of social resources, and understand how they fit into others’ functioning in the social environment. As such, when perceiving a victimizing environment triggers resource-conservation motives, we argue that the politically skilled will better understand which resources will yield the best return on their investment, and how much of those resources they need to protect. In contrast, the less politically skilled will be more likely to engage in resources exchanges with lesser returns, forcing them to focus on conserving their resources at the expense of engaging in performance exchanges.

Political skill, however, does not end with astuteness, it also enables behavior and as such is considered the “mechanism through which goal-directed behavior is activated in pursuit of interpersonal objectives and/or outcomes achievement” (Ferris et al., 2007, p. 300). Under high perceptions of a victimizing environment, the politically skilled will excel in acting on their social astuteness by altering their interpersonal influence behavior both in the short-term to leverage performance-relevant resource exchanges without opening themselves to threat, and also in the long-term to gather resources to minimize continuing or new threats. To the latter point, task performance (Treadway et al., 2013) can help individuals accrue the rewards and outcomes necessary to bolster their social resources, and is positively associated with increased political skill (Ferris et al., 2005; Jawahar et al., 2008). Their capacity to appear sincere can also stop perceptions of threat from forming in the minds of others, thus reducing the likelihood of triggering defensive behavior in others who may be perceiving a victimizing work environment.

The role of political skill in the social influence process is reviewed and explored in recent work by McAllister et al. (2016). Based on existing empirical and conceptual literature, McAllister et al. (2016) suggest that political skill enhances the social influence process in three stages: opportunity recognition, opportunity evaluation, and opportunity capitalization. The social astuteness and networking ability of politically skilled workers helps them become more socially embedded, which in-turn leads to more opportunities to influence co-workers. After assessing the goals and power of themselves and their influence targets, as well as the risks of influence attempts, politically motivated workers often decide to enact political behavior in-line with their goals; their capacity for interpersonal influence and apparent sincerity helps them convince targets to believe that politically skilled workers genuinely want the best for them, which enhances the likelihood of the targets complying with the influence attempts. This process is iterative, meaning that it allows the politically skilled to build social resources such as reputation, power, and status, which then enhances their social embeddedness, heightens their own power relative to influence targets (which can reduce the risk of influence attempts), and widens the list of viable influence behaviors from which they can choose.

In the case of perceiving a victimizing environment, the political motivations of the politically skilled individuals will likely be self-serving (for review see: Kapoutsis et al., 2015) and two-fold: (1) preserve their resources and accrue more social resources, and (2) maintain performance for the sake of typical organizational rewards, and because performance loss could impair one’s reputation or status and open them up to bullying from others. As an example, politically skilled workers perceiving victimization in their work environment are likely to first consider the many relationships they’ve built, and identify those who may be easier to influence in present circumstances (e.g., those who already respect them, those who are similar, those who are not politically skilled, etc.); their astuteness and networking ability heightens the accuracy of their perceptions, and the number of people to which they have access. They then select appropriate tactics, such as ingratiation (e.g., offering favors, helping) or inspiration, to convince those appropriate targets to act in ways that align with the politically skilled worker’s preservation goals (e.g., facilitate helpful interactions, build cohesion, trust, etc.) and performance goals (e.g., contribute resources to their task completion, make decisions favoring them, provide information); their interpersonal influence and apparent sincerity enhance the likelihood that influence targets feel good about complying with the politically skilled workers. Successful influence not only enhances performance, in this example, but builds respect or trust from others (or some other sort of social resource, like reputation), which deters less resource-rich peers from bullying the politically skilled (for fear of consequences or high risk), or allows the politically skilled to successfully retaliate (by influencing others) and regain lost resources in the event of actual victimization.

Having both the understanding and means to engage in social interactions despite perceiving a victimizing environment, politically skilled employees should be more likely take advantage of those interactions to generate new resources via job performance than less skilled employees who see them as risker and focus more on managing their stress. It is important to note that perceptions of a victimizing environment function at the employee-level, meaning that even if others do not perceive a victimizing environment, the politically skilled will still be motivated leverage their resources and enhance their performance.

*Hypothesis 1:* Political skill and perceptions of a victimizing environment will interact to predict task performance, such that employees with low political skill will exhibit reduced task performance when perceptions of a victimizing environment are high, while those with high political skill will not.

## Study 1

### Sample and Method

The sample for Study 1 was drawn from a regional United States franchise of an international food service chain. Employees often rotate between a number of formalized and standardized tasks to deliver consistent service to customers, as such, they are granted little freedom for improvisation in their task structure beyond addressing customer issues. Being customer-facing, employees must abide by set display rules regarding their emotional expressions to their customer (and each other when customers can observe them), thus inducing stress known as emotional labor (Hochschild, 1983; Morris and Feldman, 1996). While not debilitating, we expect that employees in this sample experience higher levels of emotional labor than those in less service-based contexts, perhaps depleting their resources and leaving them more vulnerable to stressors such as a victimizing environment. Task performance were rated by supervisors 3 months after victimization and political skill were measured for the purpose of reducing common method bias. Of 494 employee surveys distributed, 100 were returned (20.2% response rate), 86 contained usable longitudinal data on task performance. Supervisor ratings of task performance were acquired from 23 supervisors, with an average of 3.74 subordinates each. The sample was 79.1% female, with on average 29.4 years of age and 2.3 years tenure.

The following methods were approved by a university-associated institutional review board, including an organizational sponsorship letter signed by a high-ranking organizational representative in management agreeing that the organization will follow the approved protocol and will not pressure their employees into participating, nor will they seek to obtain individually identifying responses from the researchers. Once agreed and approved, employees were notified via either email or word-of-mouth from managers that a study would be taking place. Each employee in the sampling frame was sent a personally addressed envelope with a consent form, prize raffle ticket (random drawing for gift cards), pre-paid business reply envelope addressed to the researchers, and survey with a uniquely generated code number printed on the top right corner. Participants were encouraged to complete the survey in private, and return to the researchers from a non-work-site mailing location. A nearly identical process was followed in collecting time 2 performance surveys. Once collected, the performance data was matched to the employee response data, and names were deleted (leaving only code numbers).

### Measures and Data Analysis

#### Perceptions of a Victimizing Environment

Perceptions of a victimizing environment were measured by employees as the perceived frequency of victimizing incidents committed by others against themselves or their peers. Eight items measured perceptions of a victimizing environment for all three studies (α = 0.86) using a modified Aquino et al. (1999) measure and a 5-point Likert-type scale (1 = never, 5 = 10 or more times). Example items include: “said bad things about you or your coworkers,” “sabotaged the work of you or your coworkers,” and “did something to make you or your co-workers look bad.” Given that the item wording was only changed in terms of the referent (i.e., employees *and* their co-workers) rather than the active content of the items, threats to changes measurement validity are minimized.

#### Political Skill

Political skill was measured with the 18-item Political Skill Inventory (Ferris et al., 2005) in all studies (α = 0.89). Employees reported their agreement with items about themselves on a 7-point Likert-type scale (1 = strongly disagree, 7 = strongly agree). Example items include: “I am particularly good at sensing the motivations and hidden agendas of others,” and “I am able to communicate easily and effectively with others.”

#### Task Performance

Manager ratings of performance were collected 3 months after the initial data collection. Store managers evaluated each employee on overall task performance for the previous 3 months using a 5-point Likert-type scale (1 = poor, 5 = excellent).

#### Control Variables

Due to the sample being largely female, we tested for the impact of gender on performance ratings. This test was statistically significant, see **Table 1**, and as such we opted to retain gender as a control variable to account for any potential systemic bias in performance evaluations that may have arisen from a gender-skewed environment.

**Table 1:** Means, standard deviations, and correlations among variables in Studies 1 and 2.

Variable	Mean	*SD*	1	2	3	4	5	6	7	8
(1) Gender	0.21	0.41	-						*Study 1 (N = 86)*	
(2) PVE	1.87	0.80	0.05	(0.86)						
(3) Political skill (PS)	5.60	0.67	0.04	-0.02	(0.89)					
(4) PS: Social astuteness	5.52	0.67	0.15	-0.02	0.81^∗∗^	(0.76)				
(5) PS: Interpersonal influence	5.97	0.77	0.09	-0.10	0.83^∗∗^	0.60^∗∗^	(0.81)			
(6) PS: Networking ability	5.31	0.97	-0.07	0.04	0.87^∗∗^	0.56^∗∗^	0.58^∗∗^	(0.84)		
(7) PS: Apparent sincerity	5.84	0.72	0.01	-0.05	0.63^∗∗^	0.39^∗∗^	0.60^∗∗^	0.36^∗∗^	(0.77)	
(8) Task performance (time 2)	3.67	0.90	-0.29^∗∗^	-0.01	-0.04	-0.02	0.02	-0.08	0.01	-
(1) Task performance (time 1)	2.90	0.57	-						*Study 2 (N = 217)*	
(2) PVE	1.26	0.51	0.18^∗∗^	(0.88)						
(3) Political skill (PS)	5.28	0.79	-0.08	-0.10	(0.91)					
(4) PS: Social astuteness	5.03	0.97	-0.07	-0.06	0.91^∗∗^	(0.81)				
(5) PS: Interpersonal influence	5.68	0.82	-0.01	-0.17^∗^	0.79^∗∗^	0.62^∗∗^	(0.75)			
(6) PS: Networking ability	5.07	0.95	-0.09	-0.08	0.91^∗∗^	0.76^∗∗^	0.61^∗∗^	(0.78)		
(7) PS: Apparent sincerity	5.57	0.83	-0.12	-0.07	0.79^∗∗^	0.67^∗∗^	0.59^∗∗^	0.63^∗∗^	(0.69)	
(8) Tension	2.73	0.73	-0.08	0.23^∗∗^	-0.03	0.03	-0.12	0.00	-0.08	(0.75)
(9) Task performance (time 2)	2.77	0.56	0.30^∗∗^	-0.01	0.01	0.06	-0.05	0.01	0.00	-0.19^∗∗^

*PVE, perceptions of a victimizing environment; ^∗^*p* < 0.05, ^∗∗^*p* < 0.01*.

In terms of levels-of-analysis, this study examined perceptions of a victimizing environment at the employee-level. Although, *r*_wg(j)_ agreement measures with a rectangular null distribution were within acceptable ranges (e.g., James et al., 1984; Glick, 1985; Bliese, 2000) for groups with more than three members (supervisor-based groups, *r*_wg(j)_ = 0.85), reliability measures (e.g., ICC1, ICC2) were not (ICC1 = -0.07, *p* > 0.10, ICC2 = -0.61). From this we determined that perceptions of a victimizing environment were not appropriate for aggregation to the group climate level. Next, job performance ratings did not vary significantly between supervisors (ICC1 = 0.002, *p* > 0.10; ICC2 = 0.04; Bliese, 2000), and so were analyzed using simple moderation with predictor mean-centering (Aiken and West, 1991).

Finally, the sample for Study 1 was drawn from the United States, and the majority of studies published using samples in the United States or countries with similar norms suggest that politically skilled individuals in those countries exhibit adaptive behavior that leads to better-than-average performance when faced with stressors, and/or those low in political skill exhibit worse-than-average performance, not vice-versa (e.g., Hochwarter et al., 2007; Blickle et al., 2008, 2009; Andrews et al., 2009; Ferris et al., 2009; Kapoutsis et al., 2011). As such, if our interaction results when testing Hypothesis 1 were statistically significant, we expected them to be in that direction, and as such used only a one-tailed test of simple slopes; this also enhanced statistical power in Sample 1, which was smaller than Sample 2.

### Results and Discussion

Means, standard deviations, and correlations appear in the upper section of **Table 1**, and regression results appear in **Table 2** (only standardized beta coefficients are displayed). Mean levels of perceiving a victimizing environment were around the “one to three times” in the last year anchor point, suggesting that victimization is present in this environment, yet not widespread. As expected, there is no statistically significant direct effect of perceiving a victimizing environment on task performance, meaning that the relationship may be difficult to interpret without considering the moderating effect of political skill. Interestingly, political skill itself did not correlate with task performance, nor did any of its four dimensions. Although past research has found a direct link between political skill and performance (e.g., Ferris et al., 2005), it is possible that political behavior is less common in this particular work context, which would reduce the frequency of political skill usage. Employees in this sample work with highly formalized procedures and centralized management that are designed to maintain the consistency of product served to customer, research on perceptions of politics indeed finds that formalized work can inhibit the formation of politics perceptions (for review see: Ferris et al., 2002), which may reduce the likelihood of employees either being motivated to behave politically or to engage their political skill. The presence of victimization perceptions, however, appears to provide such motivation, and facilitates the link from political skill to performance (see results below). Finally, although all dimensions significantly correlated with political skill, apparent sincerity exhibited the lowest magnitude compared to the other three. It is conceivable that the high emotional labor demands of employees in this sample reduces variation in appearing genuine, thus reducing its convergence with the other dimensions of political skill.

**Table 2:** Multiple regression results for task performance in Study 1.

Variables	Supervisor-rated task performance (study 1)		
	Model 1	Model 2	Model 3
	β	β	β
Gender	-0.29^∗∗^	-0.29^∗∗^	-0.33^∗∗^
Perceptions of a victimizing environment (A)		0.01	0.01
Political skill (B)		-0.02	-0.05
Interaction (A × B)			0.26^∗∗^
*F*-value	7.82^∗∗^	2.57	3.65^∗∗^
*R*^2^	0.09	0.09	0.15
Adjusted *R*^2^	0.07	0.05	0.11
Δ*R*^2^	0.09^∗∗^	<0.01	0.07^∗^

*Study 1, *N* = 86; ^∗^*p* < 0.05, ^∗∗^*p* < 0.01*.

Confirmatory factor analysis was conducted to assess the discrimination between political skill and victimization. Our *p:r* ratio of 9 (variables to needed factors) and *N:p* ratio of 3.18 (sample size to variables) indicate moderate to poor appropriateness for factor analysis along traditional referents (MacCallum et al., 1999), and the combination of our low communality for items between the two latent factors (mean communality = 0.40, most items were in the range of 0.25-0.50), our *p:r* value, and sample size indicate a moderate risk of convergence issues and inadmissible solutions (MacCallum et al., 1999). Further, relative to the average factor loadings with all items (mean standardized aaa = 0.61), our sample size is below the minimum recommended for a 2-factor model with 8+ indicators per factor (Wolf et al., 2013). To address these issues, we bundled the items for political skill into their four dimensions, and randomly assigned the victimization items into three bundles (Bagozzi and Edwards, 1998). The mean standardized aaa increased to 0.76 in the bundled analysis, greatly reducing the risk of parameter bias. Fit of this model was acceptable (χ^2^ = 17.26, *df* = 13, *p* = 0.19; RMSEA = 0.06, 90CI: 0.00, 0.13; CFI = 0.98; SRMR = 0.04), and better than the fit of a one factor model (χ^2^ = 132.41, *df* = 14, *p* < 0.01; RMSEA = 0.31, 90CI: 0.27, 0.36; CFI = 0.48; SRMR = 0.19; χ^2^_diff_ = 115.15, df_diff_ = 1, *p* < 0.01). We now move on to hypothesis testing.

**FIGURE 2:**
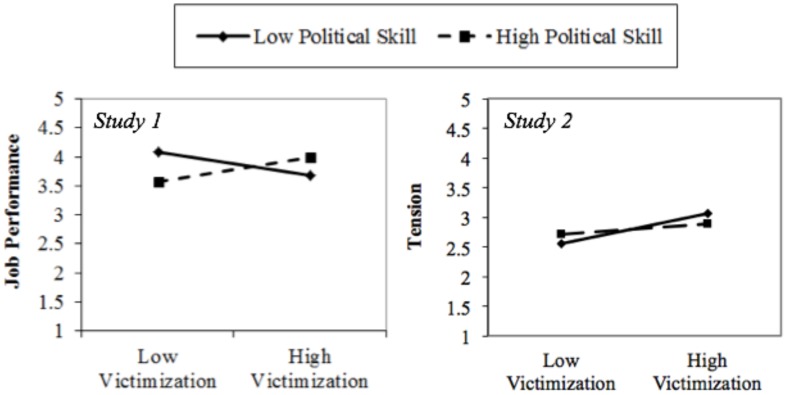
**Outcomes on the interaction between perceptions of a victimizing environment and political skill for Studies 1 and 2**.

As predicted in Hypotheses 1, the interaction of perceptions of a victimizing environment and political skill significantly affects task performance (**Table 2**) rated longitudinally by supervisors. Simple slopes analysis reveals a significant negative slope on task performance (slope = -0.25, one-tailed *p* < 0.05) for employees low in political skill, providing support for Hypothesis 1. Interestingly, however, this analysis (**Figure 2**) also reveals a significant positive slope on task performance (slope = 0.27, one-tailed *p* < 0.05) for employees with high political skill, suggesting that not only did political skill neutralize the negative effects of perceiving a victimizing environment in this sample, but it actually *enhanced* performance in highly politically skilled employees beyond that in environments with low victimization. We will address this unexpected result in the general discussion section after presenting the results of Study 2, but see these results overall as still supportive of Hypothesis 1.

The results of Study 1 offer initial evidence to support our hypothesis that politically skilled individuals are able to avoid the performance detriments associated with perceiving a victimizing environment. There are, however, two major limitations of Study 1. First, the survey response rate for Study 1 was below the mean rate observed in organizational research, which is estimated at roughly 35% (Baruch and Holtom, 2008), and may even only be somewhere between the 10th and 20th percentile of study response rates (Anseel et al., 2010). Such a low response could potentially indicate a lack of external generalizability. Second, our hypothesizing relied on the operation of COR theory, with which we argued that politically skilled individuals are less likely to experience the stress associated with social resource depletion, yet we did not include any measure to test that mechanism. In light of these limitations, we conducted a second study. In Study 2, we included a stress-based mediator as a more thorough test of the COR model, and sought a higher response rate in a new organization. As pointed out by Rogelberg and Stanton (2007), a high response rate does not equate to low non-response bias, yet replication with new methods, however, is an effective means of addressing such potential bias. As such, we also sought to replicate the findings using of Study 1 a more complex and explanatory analysis technique, regression-based moderated-mediation.

## Study 2

### Evidence for COR Effects via Replication Test

As discussed earlier, according to COR theory, actual resource loss, the threat of resource loss, or impaired recovery of resources each elicits a stress response. Due to their capacity to better manage their social resources in the face of what they perceive is a victimizing environment, the stress response of politically skilled workers should decrease. Whether rooted in their confidence and sense of control (c.f., Perrewé et al., 2004), their ability to determine high-yield resource exchanges, or their capacity to strategically accrue resources in the long-run, reduced stress should buffer politically skilled employees from the tension and strain reactions that can more enduringly impair their functioning. This reduction in tension could further protect their ability to leverage their social resources for the sake of performance rather than protection, and would indicate the functioning of COR within the perceptions of a victimizing environment-job performance link. As referred to earlier, indicators of resource depletion, such as the experience of tension or related states (e.g., emotional exhaustion) can impair task performance through two routes: (1) workers feeling like their organization failed to take care of them, so they stop reciprocating via performance (e.g., Wright and Cropanzano, 1998; Cropanzano et al., 2003), or (2) workers become fatigued and exhibit worse performance (c.f., Wright and Bonett, 1997).

*Hypothesis 2:* Political skill will moderate the indirect effect of perceiving a victimizing environment on task performance via tension, such that perceptions of a victimizing environment will negatively predict task performance via tension for employees with low political skill, yet not for employees with high political skill.

### Sample and Method

The sample to test Study 2 was drawn from the Mexican branch of a large and internationally recognizable parcel service company. Employees engage in highly formalized and standardized work behavior to coordinate and enact the delivery of parcels to customers. There is, however, little customer interaction on the part of most employees, as such we do not expect the employees to experience emotional labor beyond that for a normal environment. Employee performance was rated by supervisors via the Human Resources department in the years before and after the study took place; perceptions of a victimizing climate, political skill, and tension were measured 3 months before the second performance evaluation cycle. All survey measures were translated into Spanish using methods outlined by Brislin (1970). Measures of victimization perceptions, tension, and performance are basic and easily understood concepts, which we expect to have consistent meaning across cultures, and political skill, although more complex, has also been found to function consistently in different cultural contexts (Lvina et al., 2012). Of 667 employee surveys distributed, 288 were returned (43.2% response rate), and 217 contained usable longitudinal data. One missing value was found in the measure of tension, but was imputed using full information maximum likelihood during analyses (all analyses were conducted using Mplus; Muthén and Muthén, 1998-2012). The sample was 100% male, with an average 33.7 years of age and 3.7 years tenure in the organization.

### Measures and Data Analysis

Measures for perceptions of a victimizing environment (α = 0.88) and political skill (α = 0.91) were the same as in Study 1. Tension was measured using House and Rizzo’s (1972) 6-item job tension scale in Sample 2 (α = 0.75 after item removal, see below) on a 5-point Likert type response range (1 = strongly disagree, 5 = strongly agree). Example items include: “My job tends to directly affect my health,” and “I often feel fidgety or nervous as a result of my job.” Lastly, manager ratings of task performance were collected from archival data the year before the study and again a few months after data collection was completed. Unlike Study 1, only age held a statistically significant correlation with the dependent variables (tension, performance), not gender or tenure, and as such was retained as a control variable.

Moving on to analytical concerns, first, due to the archival nature of the performance measures, the individual identities of supervisors within each work site location were not available for each employee with supervisor-rated performance. As such, tests for group-level bias in supervisor ratings were assessed at the work site location-level. Supervisor ratings of performance did vary significantly (ICC1 = 0.23, *p* < 0.01, ICC2 = 0.88) across locations (*k* = 25 locations, mean group size = 8.68), and as such statistical analyses were conducted using multi-level path analysis to control for location-level differences in performance ratings. Second, perceptions of a victimizing work environment showed acceptable *r*_wg(j)_ agreement, using a rectangular null distribution, for groups with more than three members (location-based groups, *r*_wg(j)_ = 0.95), yet not acceptable reliability measures (ICC1 = 0.02, *p* > 0.10, ICC2 = 0.24). Once again, we found no evidence to suggest the perceptions of a victimizing environment could be aggregated to the group-level. Finally, in contrast with the sample in Study 1, our sample in Study 2 was drawn from Mexico, which tends to exhibit greater power distance and less individualism in its culture than does the United States (Hofstede, 1980). Little work has been conducted on the interactive effects of political skill and work perceptions as they affect performance in Mexican samples, so we opted for two-tailed tests in our simple slopes analysis.

### Results

**FIGURE 3:**
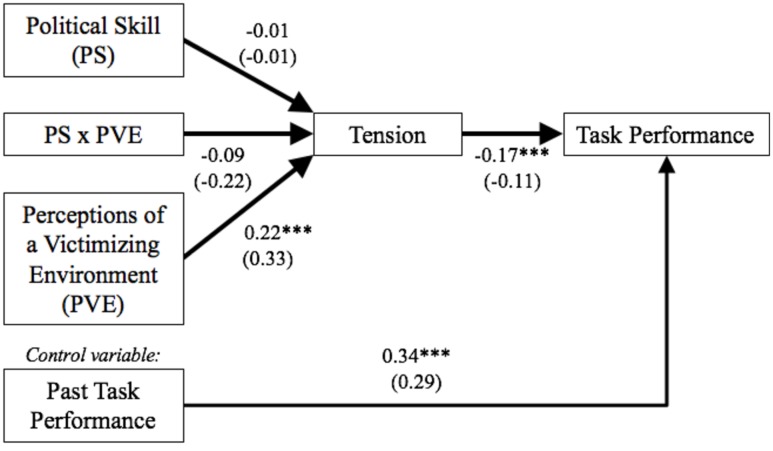
**Multi-level path analysis results for Study 2**. *N* = 217, unstandardized parameter estimates are shown in parentheses, multi-level modeling to remove the effects of location-differences on task performance evaluations not shown; ^∗∗∗^*p* < 0.001.

Means, standard deviations, and correlations appear in the lower section of **Table 1**, and multi-level path analysis results in **Figure 3**. As expected, perceptions of a victimizing environment positively correlated with tension, which then negatively correlated with task performance. Next, the dimensions of political skill more strongly correlated with one another than in Study 1. Because the work conducted in Study 2 involves less customer interaction, it likely has weaker norms for emotional labor, which offers minor evidence to confirm our suspicion than emotional labor-intensive environments are linked with forced apparent sincerity. Political skill once again did not correlate with task performance, which is again surprising, but may also be due to the formalized and centralized nature of the work in this sample. Interestingly, the interpersonal influence dimension of political skill exhibited a small negative correlation perceiving a victimizing environment; assuming others fear victimization as well, this could have arisen because workers were more suspicious and vigilant toward their co-workers’ motives, thus diminishing the ease of influencing them. Lastly, and also unexpectedly, previous year task performance was significantly and positively correlated with perceiving a victimizing environment. No conclusions can be drawn without conducting further independent study of this relationship, but it could be possible that high performers are also more vigilant to threatening behavior (and thus perceive it more readily) because they expect to be envied by others (c.f., Exline and Lobel, 1999); that untested interpretation should be read with caution.

In further testing the validity of our COR-based model, we test the conditional indirect effect of perceiving a victimizing environment on task performance via tension for employees with low political skill compared to those with high political skill. Conditional indirect effect analysis involves using the “bootstrapping” re-sampling method to create a distribution of indirect effect product coefficients against which to more accurately test our interaction hypothesis (Bollen and Stine, 1990; Shrout and Bolger, 2002; MacKinnon et al., 2004; Preacher et al., 2007). However, we conduct this analysis within a multi-level path model to remove the effects of employee location (as discussed above).

First, confirmatory latent factor analysis was conducted with political skill, victimization, and tension, with political skill items bundled into four facets based on the dimensions of political skill to better capture its dimensionality. Factor loadings were acceptable for all items except for item 2 (standardized aaa = 0.28) of the tension measure; because item 2 may not well represent assessments of tension in this particular sample, it was dropped from subsequent analyses. Results suggest the three factor model (χ^2^ = 271.69, df = 116, *p* < 0.01; RMSEA = 0.08, 90CI: 0.07, 0.09; CFI = 0.91; SRMR = 0.06) is a better fit for the data than a two factor model with perceptions of a victimizing environment and tension loading on one factor (χ^2^ = 473.40, df = 118, *p* < 0.01; RMSEA = 0.12, 90CI: 0.11, 0.13; CFI = 0.79; SRMR = 0.10; χ^2^_diff_ = 201.71, df_diff_ = 2, *p* < 0.01) or a one factor model with political skill, perceptions of a victimizing environment, and tension loading on one factor (χ^2^ = 1334.48, df = 119, *p* < 0.01; RMSEA = 0.22, 90CI: 0.21, 0.23; CFI = 0.28; SRMR = 0.24; χ^2^_diff_ = 1062.79, df_diff_ = 3, *p* < 0.01). Second, for the path analysis, first, a saturated path model is test using grand mean centering for the interaction and its component predictors (i.e., perceptions of a victimizing environment, political skill). All paths without statistically significant unstandardized parameters at an α = 0.05 level were removed. The resultant model is in **Figure 3**. This model matches the hypothesized model and fits the data very well (χ^2^ = 9.68, df = 4, *p* = 0.05; RMSEA = 0.08; CFI = 0.91; SRMR = 0.04). *R*^2^ values for the final model are as follows: task performance (time 2) = 0.14 (*p* < 0.01), and tension = 0.06 (*p* = 0.08). Tests of conditional indirect effects were conducted in MPlus.

Standardized parameter estimates reveal that perceptions of a victimizing environment significantly correlated with tension, and tension significantly predicted task performance, beyond the effects of previous year performance ratings. Conditional indirect effect analysis finds that perceiving a victimizing environment only negatively affects task performance through tension among employee with low political skill (indirect effect = -0.06, *p* < 0.05), not for those with high political skill (indirect effect = -0.02, *p* = 0.29). Further, simple slopes analysis (**Figure 2**) reveal that perceptions of a victimizing environment are only significantly and positively related to tension for employees with low political skill (slope = 0.51, *p* < 0.01), not those with high political skill (slope = 0.16, *p* = 0.23). In total, these results offer support for Hypothesis 2.

## General Discussion

Corley and Gioia (2011) assert the importance of having “prescience” in our research, by developing solutions, now, to the problems that will face employees in the future. Workplace victimization is one such problem. It has been recently recognized as a commonplace issue affecting over one-third to half of workforces across the world, and is on the rise (Chappell and Di Martino, 2006; Know Bull!, 2010; Monster.com, 2011; Workplace Bullying Institute, 2014). Given the ever-increasing financial hardships organizations face in a competitive and globalized world, as well as the heightened social connectedness between employees, it may only be a matter of time before victimization jumps to new heights or evolves as business practices advance. In exploring political skill as a potential antidote to the performance ramifications of perceiving a victimizing environment, we intended to offer new avenues to deal with this issue.

Considering that victimization is as much a social phenomenon as it is a personal one, we contended that perceptions of a victimizing environment as a whole would affect employee job performance through the social perceptions and social resources that maintain both victimization (Aquino et al., 1999; Aquino and Bommer, 2003; Aquino and Lamertz, 2004) and performance (Gerstner and Day, 1997; Sparrowe et al., 2001; Thompson, 2005). Through the lens of the COR perspective (Hobfoll, 1988, 1989, 2002), we then argued that employees’ capacity to effectively regulate their social resources, their political skill, will determine the effect of perceiving a victimizing environment on performance. Using two organizational field samples, we found evidence to support most of our assertions.

First and foremost, we found evidence in Study 1 that employees with low political skill exhibited worse task performance when they perceived a victimizing environment than when they did not. The link between perceiving a victimizing environment and task performance was mediated by increased tension, as found in Study 2, which offered further support that social resource depletion was a primary driver of the effect, as we had theorized. Second, support was also found for a non-hypothesized effect, employees with high political skill in Study 1 exhibited not only a lack of performance decrement when they perceived victimization in their environment compared to those who did not, but an *increase* in task performance when they perceived a victimizing environment. This effect was not replicated in Study 2, leaving us to suspect that it was an artifact of the unique characteristics of Study 1. Unlike employees in Study 2, whose primary tasks involved managing and delivering inventory, employees in Sample 1 were customer-facing and experience more stress to adhere to emotional expression standards (i.e., emotional labor; Hochschild, 1983; Morris and Feldman, 1996). Employees with high political skill have been found to be resilient to emotional labor when engaging in political behavior, unlike their low political skill counterparts (Treadway et al., 2005), which may have empowered them to leverage social relationships with emotionally taxed lower political skill workers, who were already struggling to combat stress from victimization, toward building more social resources and thus enhancing their performance over time.

Overall, however, our study shed further light on the role of resource management in coping with victimization-type stressors, and their effects on job performance. Unlike the politically skilled who were able to efficiently regulate their social resources, those low in political skill did not adapt as well to perceiving a victimizing environment, exhibited signs of resource depletion, and performed worse on the whole. The present research complements recent work on the capacity of political skill to preserving personal resources (e.g., confidence, etc.; Lazarus and Folkman, 1984) in response to personally targeted aggression (Zhou et al., 2015) by demonstrating its functionality in response to broader social threat and social resource management. Our primary contribution is the expansion of the victimization literature the domain of social-relational mechanisms based on the COR model, which acts as an additional pathway to performance from victimization beyond personal resources. Our second contribution is to the literature on political skill. Not only does this study suggest that political skill helps employees perform better in victimizing environments, but it also demonstrates that political skill affects the link between negative environmental perceptions and job performance.

### Theoretical and Practical Implications

At the core of our study is the idea that perceptions of a victimizing environment drain social resources, which then lowers performance for those who are unable to effectively manage such resources. This idea stands in complement to recent work by Thau and Mitchell (2010) and Lian et al. (2014) who suggest that abuse depletes self-regulatory resources (i.e., Baumeister et al., 1998), which then causes negative reactions and further deviance. Although Thau and Mitchell (2010) and Lian et al. (2014) were investigating self-control, which is a personal resource focused on personal regulation, their findings might generalize to broader regulatory processes and also encompass personal resources that facilitate social resource regulation, like political skill. Integrating our findings into the self-control failure model, would suggest that not only is social resource depletion the mechanism behind performance failure in employees with low political skill, but that such performance failure results from loss of impulse control that in turn damages social resources (e.g., emotional outburst toward valued others, abuse of status for personal gain, violating the trust of others for personal gain or relief, etc.) and thus performance.

Future research could integrate these two perspectives and examine the interaction of traits encourage self-control activation (e.g., power, rewards, proactivity, etc.; Lian et al., 2014) with the availability of social resources. Is, perhaps, self-control more useful for reducing stress in those individuals who have more social resources, or are more politically skilled enough to effectively manage such resources? While our work and that of recent others (i.e., Thau and Mitchell, 2010; Lian et al., 2014) have taken steps toward conceptualizing the role of resource regulation in predicting reactions to hostility and victimization, none have yet tested it directly. This might be methodologically challenging, but laboratory measurement of teams who are allowed to have different social resources (or measuring changes in existing teams with their own unique constellations of social resources) has the potential to shed more light upon the mechanics of social resource regulation failure in victimization.

Next, our analysis may also have ramifications for conceptualizing victimizing environments at the group-level. The low differentiation we found between groups (i.e., ICC1, ICC2) in terms of victimization climate in both samples 2 and 3 may suggest that victimizing environments are best conceived at the employee level and are not stable for long enough to easily measure. In line with COR theory (Hobfoll, 1989), it is conceivable that group members may only act in conservative ways until the climate dissolves, as managers take actions in the larger organizational system to quell hostility. If such environments are fleeting, they are still worth study, but understanding their nature and successful interventions may involve longitudinal measurement over time and more intensive types of multi-level analysis (e.g., latent growth curve modeling; Raudenbush and Bryk, 2002).

Finally, in terms of practice, our study demonstrated that political skill can moderate the relationship between a severe, aggression-based stressor and behavior, just as it has been previously linked to the weakening effects of stressors on attitudes and strain (e.g., Perrewé et al., 2004). These findings suggest that practitioners dealing with increasing perceptions of victimization may benefit from training the political skill of their employees, or interventions targeted toward helping employees better manage their social resources, such as learning about the unique personality and motives of their teammates (e.g., team-building workshops), learning to manage their reputation with co-workers and customers, or even sessions to help them personally interact with one another in ways that build or preserve trust. Although we used political skill here, others indicators of effective social resource regulation, such as emotional intelligence or receptivity to workplace socialization may function similarly and would be worthwhile for organizations to keep in mind when selecting, placing, and training employees. Some of the interventions mentioned above (e.g., team-building) might also reduce victimization perceptions by bettering co-workers relations, thus reducing the needs to boost political skill. It would likely be more ethical to attempt to stop the cause of victimization perceptions rather than only manage the reaction to them, and initiatives like culture-building, mentoring, leadership role-modeling or other norm-setting behavior may enhance the supportiveness of employee interactions. However, because we found these perceptions did not aggregate to the group-level in our two studies, we caution against interpreting one individual workers’ perceptions of victimization as representative of actual social dynamics; addressing the cause of perceiving a victimizing environment may best be handled on a person-to-person basis.

### Strengths and Limitations

First, whereas internal validity may have been even more enhanced by one large sample in a single organization, our offering of two samples offers greater confidence in the generalizability of the model, especially across United States borders. Next, our measure of perceptions of a victimizing environment was only a simple extension upon Aquino et al.’s (1999) measure, and as such only offers a behavioral frequency-based measurement of victimization in the environment. While this does preserve the features of the measure (suggesting that we have not altered the construct in ways unintended), future work would benefit from developing a unique measure of perceptions of a victimizing environment to better capture the domain. Even though we were unable to aggregate perceptions of a victimizing environment, our use of multi-level modeling keeps tests of it bound to the employee-level. Third, although fit was strong for our statistical model in Study 1, it was not as strong in Study 2. The replication of results provides some support for the factor structure in Study 2, but in light of our measurement model difficulties, we suggest that further replication is needed.

Lastly, there were two major issues that threaten the validity of our measures of performance. First, in both studies we only obtained single-item measures of performance. Although single-item measures are less than ideal, both (which were different in nature) yielded similar results regarding the basic interaction, lending some evidence for consistency and validity; moreover, research on single-item measures has found evidence that they often function no different than multi-item measures in terms of method bias and validity (e.g., Gardner et al., 1998; Bergkvist and Rossiter, 2007). Second, past research has demonstrated that the politically skilled are able to effectively leverage social influence behavior to heighten the performance evaluations from others (e.g., Harris et al., 2007), which could mean that performance ratings were biased. However, the lack of direct correlation between political skill and performance evaluations leads us to believe this is not a significant problem in our two studies. Even if it were an issue when perceiving a victimizing environment, and we were unable to detect it, performance evaluations are often used as a metric for success, and could still serve to benefit the social resources of the politically skilled (e.g., reputation, etc.), supporting our premise.

Given our attempts to compensate for the limitations of our studies, we believe this research still contributes to the advancement of the organizational sciences. With the increasingly social and connected nature of work in light of technological advancements and the changing nature of work, the utility of social resources in facilitating performance and managing stress may be reaching new heights. We hope our research serves useful to managers and professionals in preparing workers to excel in the environments, and building a healthier and more effective workplace.

## Ethics Statement

The current study was approved by the human Subjects Committee at the University at Buffalo.

## Author Contributions

JB contributed to developing the theoretical framework and justifications, data analysis, organization, and overall writing of the manuscript. DT contributed to editing and organization of the paper, as well as data acquisition and overall design. LW, BG, and JY all contributed to the design, framing, variable choice, and management of the study, as well as the process of data acquisition, and editing.

## Conflict of Interest Statement

The authors declare that the research was conducted in the absence of any commercial or financial relationships that could be construed as a potential conflict of interest.
